# Prognostic impact of the lymph node yield on survival in patients with stage I lung adenocarcinoma receiving sublobar resection

**DOI:** 10.3389/fmolb.2026.1727569

**Published:** 2026-03-24

**Authors:** Yongzhong Li, Jiangtao Li, Xuejun Xu, Qifeng Ding, Yongbing Chen, Yiming Mao

**Affiliations:** 1 Department of Thoracic Surgery, the Second Affiliated Hospital of Soochow University, Suzhou, China; 2 Department of Thoracic Surgery, Suzhou Kowloon Hospital Shanghai Jiao Tong University School of Medicine, Suzhou, China

**Keywords:** lung adenocarcinoma, lymph nodes, prognosis, sublobar resection, surgery

## Abstract

**Introduction:**

It remains controversial regarding the prognostic impact and therapeutic implications for immunotherapy of lymph node yield (LNY) during sublobar resection (SR) on stage I lung adenocarcinoma (LUAD).

**Methods:**

We analyzed a retrospective cohort of 400 patients with stage I LUAD who underwent SR, with peripheral blood samples prospectively collected for detecting inflammatory cytokines (IFCs). The effect of different LNY (≥4 vs. <4 nodes) on survival and IFC change was evaluated. Consensus clustering analyses were performed using data from The Cancer Genome Atlas (TCGA) and our validation cohort to explore associations between IFCs and immune/cell death profiles. A Bayesian meta-analysis was further conducted to assess the impact of LNY in LUAD undergoing SR.

**Results:**

The survival analysis of our cohort demonstrated that increased LNY during SR did not prolong RFS (≥4 vs. <4 nodes: HR = 1.15; 95%CI: 0.76–1.74). A lower LNY during SR was associated with significantly better RFS in stage I LUAD receiving adjuvant immunochemotherapy (≥4 vs. <4 nodes: HR = 0.41; 95%CI: 0.17–0.94). In terms of IFCs, extensive lymph node dissection led to significantly increased levels of IL-6, IL-4, IL-10 and TNF-α after SR (*p* < 0.05). Consensus clustering based on the IFCs identified two subgroups (Cluster 1 and 2) in TCGA cohort with distinct immune and cell death profiles, including differences in immunogenic cell death and damage-associated molecular patterns. Cluster 2 exhibited a higher Tumor Immune Dysfunction and Exclusion (TIDE) and tumor mutation burden scores. Similar findings were observed in our validation cohort, where Cluster 2 displayed higher number of neoantigens. The Bayesian meta-analysis also corroborated that increased LNY did not improve RFS (HR = 0.98; 95%CI: 0.20–2.94) in pathological stage I LUAD.

**Discussion:**

Increased LNY during SR might confer no additional benefits to RFS for p-stage I LUAD. Excessive removal of LNs might exert adverse impact on physical sensitivity to immunochemotherapy. Personalized lymph node management should be adopted for selected node-negative disease.

## 1 Introduction

Although lobectomy with hilar and mediastinal lymph node (LN) dissection remains the standard surgical procedure for clinical stage I non-small cell lung cancer (NSCLC). The recent multicenter randomized controlled non-inferiority trial (CALGB140503) has established sublobar resection (SR) as a viable alternative to lobectomy for small-sized NSCLC (Altorki et al., 2024). However, clinicopathological factors influencing the oncological outcomes of SR remain incompletely elucidated.

In recent years, the adequacy of LN harvest has been increasingly emphasized as a quality indicator following curative-intent resection for NSCLC (Al-Thani et al., 2024). A previous study of 3,269 patients with stage I NSCLC undergoing SR demonstrated that dissection of more than four regional LNs was associated with better survival compared with dissection of 1–3 LNs (Cao et al., 2018). Similarly, another population-based study confirmed that examining ≥3 LNs was associated with a more favorable prognosis than inadequate dissection (He et al., 2020). A National Cancer Database study also suggested that more extensive lymph node sampling benefited stage IA patients undergoing SR (Speicher et al., 2016). In contrast, Zheng et al., using the Surveillance, Epidemiology, and End Results (SEER) database, reported that harvest of ≥4 LNs did not provide additional survival advantage over 1–3 LNs in patients with NSCLC ≤1 cm undergoing SR (Zheng et al., 2020). Furthermore, surgical removal of tumor-draining lymph nodes (TDLNs) might abrogate immunotherapy-triggered tumor regressions (Fransen et al., 2018). Thus, the role of adequate LN dissection in stage I lung cancer undergoing SR remains uncertain.

This study aimed to investigate the prognostic significance of lymph node yield (LNY) during SR in stage I lung adenocarcinoma (LUAD), a major histology of NSCLC, by monitoring the perioperative inflammatory cytokines and characterizing the potential relationships between LNY and immune and cell death profiles.

## 2 Methods and materials

### 2.1 Study design and patient population

We conducted a retrospective review of medical records from patients with clinical stage IA LUAD, who underwent SR from January 2021 to March 2023 at Suzhou Kowloon Hospital Shanghai Jiao Tong University School of Medicine. The study flowchart is shown in Supplementary Figure S1. Exclusion criteria included: (I) pathologically confirmed non-invasive malignant disease; (II) conversion to thoracotomy; (III) intraoperative blood loss >300 mL; (IV) major postoperative complications (e.g., prolonged air leakage, pulmonary embolism, severe infection); (V) recent history of infections, autoimmune diseases, or concurrent malignancies; (VI) recent history of corticosterioids or immunosuppressive drugs. For patients with radiologically pure solid nodules, cervical, abdominal, and brain computed tomography (CT) scans or positron emission tomography/computed tomography (PET-CT) were also required.

Primary tumor and sampled LNs were sent for intraoperative frozen section examination. Only patients with intraoperatively confirmed N0 disease were included for perioperative inflammatory cytokine monitoring. These patients were divided into two groups (lower vs. higher LNY) based on the median number of LNs in our cohort. Clinical and pathological data were extracted, including sex, age, smoking history, surgical procedure, LNY, the pathologic TNM stage, operation time (OT), intraoperative blood loss (IBL), pleural effusion drainage volume (PEDV), thoracic drainage tube retention time (TDTRT), and postoperative hospital-stay (PHS), adjuvant therapy, immunotherapy regimens for recurrence controlling, treatment cycle number, and recurrence information. All chest CT scans were retrospectively examined to determine the radiological features of the resected lesions. Ground-glass component was defined on a CT scan by hazy increased opacities in the lung parenchyma, with preservation of bronchial structures and vascular margins (Zhang et al., 2020). Furthermore, the PD-L1 expression levels in the resected LUAD were categorized as follows: strongly positive (≥50%), weakly positive (1%–49%), or negative (<1%).

For pathological stage IB LUAD with high-risk factors (e.g., lymphovascular invasion, wedge resection, visceral pleural involvement, high-grade predominance, or solid component ratio ≥20%), adjuvant therapy was recommended in accordance with the National Comprehensive Cancer Network (NCCN) guidelines (Riely et al., 2024) and multidisciplinary evaluation. First-line treatment with PD-1 inhibitors (pembrolizumab, nivolumab, toripalimab, tislelizumab, camrelizumab, or sintilimab) combined with platinum-based doublet chemotherapy was administered every 21 days to those patients without driver mutations, such as epidermal growth factor receptor (EGFR), anaplastic lymphoma kinase (ALK), or c-ROS proto-oncogene 1- receptor tyrosine kinase (ROS1) genetic mutations, regardless of programmed cell death 1 ligand 1 (PD-L1) expression. This study was approved by the Institutional Review Board of Suzhou Kowloon Hospital Shanghai Jiao Tong University School of Medicine (KY-2023–006).

### 2.2 LN dissection and pathological assessment

All surgeries were performed using a standardized approach by certified and experienced surgeons. Resected LN specimens were sent for pathological evaluation. Pathological assessment for resected LNs was conducted independently by at least two senior pathologists (Jiang X. and Zhao J.). Pathologists also employed a rigorous process for screening LN fragments based on LN size and boundaries. And calculation of the LN number only included structurally intact LNs and those composed of identifiable fragments. Meanwhile, the grading of lung adenocarcinoma differentiation was performed by two senior pathologists. In accordance with the 2021 World Health Organization Classification of Thoracic Tumors and International Association for the Study of Lung Cancer (IASLC) recommendations, tumor grading was defined by the predominant growth pattern and the proportion of high-grade components: Grade 1 (well-differentiated) was defined as lepidic-predominant with <20% high-grade components; Grade 2 (moderately differentiated) as acinar- or papillary-predominant with <20% high-grade components; and Grade 3 (poorly differentiated) as presence of ≥20% high-grade components (including patterns such as solid, micropapillary, or cribriform).

### 2.3 Observation indicators

Venous blood samples of patients who met the inclusion criteria and underwent intentional SR were prospectively collected on the morning before surgery to detect inflammatory cytokine indices as described in previous studies (Neff et al., 2022; riukhovetska et al., 2021; Hänggi et al., 2024; Ibusuki et al., 2024; LaMarche et al., 2024), with a sample volume of 5 mL. Blood samples were subjected to protein chip technology for detecting inflammatory cytokines (IFCs) before surgery, which were also collected on the 1st postoperative days (POD1) with IFCs tested. The methods and steps were carried out in strict accordance with the kit instructions (QAH-INF-1, RayBio® Human Inflammatory Cytokine Antibody Array 1, United States): (1) The samples were collected using untreated collection tubes and centrifuged within 24 h. Following this, the supernatant was stored at −80 °C for subsequent analysis of IFCs, which was performed within 1 month. (2) The chip was blocked and incubated, followed by the addition of 0.1 mL of 2-fold diluted serum. (3) Biotin antibody labeling was performed. (4) Chemiluminescence imaging analysis was performed using the InnoScan 300 Microarray Scanner (Innopsys, France). The measured IFCs included IL-6, IL-10, IL-8, IL-4, IL-1α, IL-1β, IL-13, MCP-1, INF-γ, and TNF-α. Comparisons were carried out using the t-test or Mann–Whitney U test according to whether the data followed a normal distribution. A *p* value <0.05 was considered statistically significant.

### 2.4 TCGA dataset and survival analysis

Transcriptomic and survival data of The Cancer Genomic Atlas (TCGA) dataset were obtained, with baseline characteristics summarized in Supplementary Table S1. FPKM-normalized gene expression values and survival information of 464 LUADs were downloaded using the R package “TCGAbiolinks” (Colaprico et al., 2016) to examine the prognostic value of the IFCs. The “ConsensusClusterPlus” R package (Guinney et al., 2015) was used to perform consensus clustering and to categorize the LUAD dataset into two clusters based on the IFCs. The analysis was configured with a maximum of 5 clusters and 500 repetitions, using a sample sampling ratio of 0.8 and a gene sampling ratio of 1. Kaplan-Meier plots were generated using R packages “survival” and “survminer”.

### 2.5 Bulk RNA-sequencing and exome sequencing

Twenty-four primary LUAD tissues were subjected to next-generation sequencing (NGS) and whole exome sequencing (WES) (Supplementary Table S2). RNA was extracted from these fresh tissues using the DNA/RNA kit. The RNA library preparation workflow comprises several steps: (1) RNA enrichment, achieved through rRNA removal using a probe-based method; (2) RNA fragmentation and random primer binding; (3) first-strand cDNA synthesis using reverse transcriptase lacking RNase H activity; (4) second-strand cDNA synthesis; (5) end repair, dA tailing, and adapter ligation; and (6) PCR enrichment using a high-fidelity polymerase to amplify and select adapter-bound molecules.

### 2.6 Endpoints and follow-ups

The endpoint was recurrence-free survival (RFS), defined as the time from surgery to local or distant recurrence. Local recurrence was defined as tumor recurrence in the ipsilateral lung or LNs, with distant metastasis referring to recurrence in the contralateral lung or LNs, or distal organs such as the brain, liver, or bone. Recurrence was diagnosed on the basis of physical examination and/or imaging findings, and the diagnosis was histologically confirmed when clinically feasible. Follow-up data were gathered using electronic care records, institutional databases, and telephone call. All patients were regularly followed up until 30 May 2025. During follow-up period, censoring events occurred due to loss to follow-up or death from non-cancer-related causes. The Kaplan-Meier method was used to estimate the recurrence rate, thereby reducing the impact of censored data on the accurate estimation of the true recurrence risk.

### 2.7 Statistical analyses

Statistical Packages for R (version 4.3.2 for Windows) was employed to conduct the statistical analyses. Variables were compared using the t-test or Mann–Whitney U test. Tumor Immune Dysfunction and Exclusion (TIDE) scores (Jiang et al., 2018) were calculated through online tools on the official website (http://tide.dfci.harvard.edu), which predicted response to immune checkpoint blockade. Tumor mutation burden (TMB) analysis (Mayakonda et al., 2018) was performed for every sample using the “maftools” algorithms. We calculated activity estimates of damage-associated molecular patterns (DAMPs) (Liu et al., 2024) and cell death patterns (Zou et al., 2022; Qin et al., 2023) for each tissue using “gsvaParam” algorithms of the GSVA package (version 1.42.0) (Hänzelmann et al., 2013). Meta-analysis was two-sided, and the general data analyses were conducted by using “R2jags”, “bmeta” and “metafor” R packages (Mostafaei et al., 2020; Röver et al., 2021). Those relevant studies were identified by searching databases including PubMed, EMBASE, Web of Science and Cochrane Library up to March 2025 without language restrictions. The hazard ratio (HR) with the corresponding 95% confidence interval (CI) was extracted from the studies which was uniformly adjusted as higher/lower LNY according to the median value of the included studies. The τ^2^ statistic was used to assess heterogeneity, with lower values indicating lesser heterogeneity. Subgroup analyses of the associations between LNY and RFS were performed which were stratified by different demographic or clinical characteristics. Intergroup and intragroup comparisons were carried out using the independent sample t-test. A *p* value <0.05 was considered statistically significant.

## 3 Results

### 3.1 Patient characteristics

A total of 400 patients were included in the analysis. Using 4 LNs as the cutoff point, there were 186 patients in the lower LNY group (<4 nodes), while 214 in the higher LNY group (≥4 nodes). Notably, this cutoff has been consistently used in a number of studies as a prognostic threshold for LNY in SR (Zheng et al., 2020; Ajmani et al., 2018; Hao et al., 2021; Huang and Petersen, 2024). The baseline characteristics of the included patient cohort were listed in Table 1. The median follow-up time was 32.6 months for entire cohort, with 68 patients lost to follow-up. Among them, 61 could not be contacted due to changed phone numbers or addresses, and 7 died from non-cancer-related causes. No statistical differences were observed in terms of gender, age, smoking history and stage between the two groups (*p* > 0.05). However, patients in the lower LNY group had significantly decreased IBL, OT and TDTRT than those in the higher LNY group (*p* < 0.05). Of note, all patients in the higher LNY group underwent segmentectomy. No significant differences were found in PEDV and PHS between the two groups (*p* > 0.05). 124 (31.0%) patients with stage I LUAD received adjuvant combination therapy, with sintilimab in combination with platinum administrated (Table 1). Their median follow-up time was 32.5 months. Notably, the lung adenocarcinomas were predominantly moderately and poorly differentiated within this cohort.

**TABLE 1:** Baseline characteristics of the patient cohort with stage I LUAD undergoing sublobar resection with different extent of lymph node dissection (cutoff: 4).

Variables	The entire cohort (n = 400)			The cohort receiving adjuvant therapy (n = 124)		
	Lower LNY (<4 nodes) (n = 186)	Higher LNY (≥4 nodes) (n = 214)	*p*	Lower LNY (<4 nodes) (n = 61)	Higher LNY (≥4 nodes) (n = 63)	*p*
Age (years)	63.1 (44∼80)	62.6 (47∼79)	0.48	65 (49∼79)	60 (47∼74)	0.02
Gender (male/ female)	96/90	114/100	0.52	36/25	32/31	0.36
Smoking history (yes/no)	86/100	78/136	0.05	30/31	19/44	0.03
Operation (wedge/seg)	52/134	0/214	<0.001	19/42	0/63	<0.001
LN dissection	2 (0∼3)	6 (4∼13)	<0.001	2 (1∼3)	6 (4∼13)	<0.001
Pathological stage (IA/IB)	103/83	129/85	0.32	6/55	7/56	0.82
Radiological features (GGN/pure solid)	80/106	70/144	0.03	13/48	13/50	0.93
Tumor differentiation (G1/G2/G3)	109/36/41	131/41/42	0.45	0/20/41	0/21/42	0.59
PD-L1 expression (strongly/weakly/negative)	14/58/114	19/62/133	0.94	9/52/0	17/46/0	0.09
IBL (mL)	145 (20∼300)	157 (30∼300)	<0.001	159 (30∼300)	164 (50∼300)	0.48
OT (h)	2.73 (1∼4.17)	2.92 (2∼4.5)	0.11	2.66 (1.50∼3.38)	2.84 (2.0∼3.83)	0.27
TDTRT (d)	4 (2∼7)	5 (3∼8)	<0.001	4 (3∼7)	5 (3∼10)	0.91
PEDV (mL)	752 (200∼1080)	851 (360∼1350)	0.45	790 (280∼1080)	837 (360∼1340)	0.44
PHS (d)	5 (3∼8)	6 (3∼11)	0.40	5 (3∼8)	6 (3∼11)	0.46
Adjuvant therapy (yes/no)	61/125	63/151	0.63	61/0	63/0	0.09
Follow-up (days)	983 (693∼1160)	985 (818∼1163)	0.89	973 (693∼1155)	982 (818∼1163)	0.63

Abbreviations: LUAD, lung adenocarcinoma; LNY, lymph node yield; GGN, ground glass nodule; seg, segmentectomy; G1, Grade 1 (well-differentiated); G2, Grade 2 (moderately differentiated); G3, Grade 3 (poorly differentiated); IBL, intraoperative blood loss; OT, operation time; TDTRT, thoracic drainage tube retention time; PEDV, pleural effusion drainage volume; PHS, postoperative hospital-stay.

Footnote: In the “Tumor differentiation (G1/G2/G3)” column, the notation “109/36/41″corresponds to 109 G1, 36 G2, and 41 G3 cases, respectively, in the low LNY, group; other entries follow the same convention.

### 3.2 Prognostic impact of LNY on survival

**FIGURE 1:**
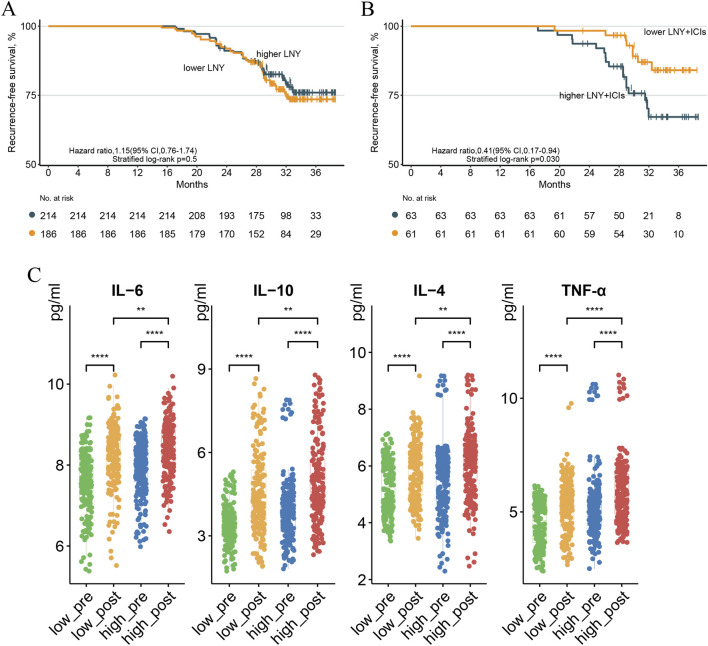
The recurrence-free survival of the higher (≥4 nodes) and lower (<4 nodes) LNY groups with p-stage I LUAD **(A)**. The recurrence-free survival of the higher (≥4 nodes) and lower (<4 nodes) LNY groups receiving adjuvant immunochemotherapy in p-stage I LUAD **(B)**. The levels of immune-related molecules in peripheral blood before and after surgery **(C)**.

During the follow-up period, 88 cases of recurrence were documented. The survival analysis by different LNY indicated a higher LNY might confer no additional benefits to RFS (HR = 1.15; 95%CI: 0.76–1.74, *p* = 0.50) in the overall cohort of patients with p-stage I LUAD (Figure 1A; Supplementary Figure S2A). However, among patients receiving adjuvant PD-1 inhibitors, a lower LNY during SR was associated with significantly improved RFS (HR = 0.41; 95%CI: 0.17–0.94, *p* = 0.03) (Figure 1B). Notably, this association remained significant in the segmentectomy-only subgroup (HR = 0.36; 95%CI: 0.13–0.99, *p* = 0.04; Supplementary Figure S2B). Multivariable Cox analysis of the 124 patients receiving immune checkpoint inhibitors (ICIs) further demonstrated that fewer LNs harvested during SR was an independent favorable predictor of RFS (HR = 0.279; 95%CI: 0.100–0.778, *p* = 0.015) with confounding variables adjusted (Table 2).

**TABLE 2:** Multivariable Cox regression of predictors for recurrence-free survival in stage I LUAD receiving adjuvant immunochemotherapy (n = 124).

Variables	HR (95% CI)	p
Age		
<65	Reference	
≥65	1.380 (0.562, 3.388)	0.483
Pathological stage		
IA	Reference	
IB	0.507 (0.155, 1.653)	0.260
Operation		
Segmentectomy	Reference	
Wedge resection	1.226 (0.289, 5.200)	0.783
LNY		
High (≥4 nodes)	Reference	
Low (<4 nodes)	0.279 (0.100, 0.778)	0.015
Radiological features		
GGN	Reference	
Pure solid	1.107 (0.316, 3.881)	0.873
Tumor differentiation		
Moderate	Reference	
Poor	0.726 (0.240, 2.196)	0.570
PD-L1 expression		
Strong	Reference	
Weak	4.951 (1.118,21.917)	0.035

Abbreviations: LUAD, lung adenocarcinoma; LNY, lymph node yield; GGN, ground-glass nodule; HR, hazard ratio.

### 3.3 Differences in IFCs based on the extent of LN dissection

No significant differences in the levels of the ten IFCs were observed between the lower LNY and higher LNY groups before surgery. IL-6, IL-10, IL-4 and TNF-α levels in both groups were significantly increased at POD1 compared with the levels before surgery (*p* < 0.05), with a more pronounced increase observed in the higher LNY group compared to the lower LNY group (p < 0.05) (Figure 1C). Levels of the other six IFCs were also significantly elevated at POD1 compared to preoperative levels in both groups (p < 0.05), but were not significantly different between the two groups (*p* > 0.05) (Supplementary Figure S2C).

### 3.4 IFCs-based profiling of distinct prognoses of LUADs

**FIGURE 2:**
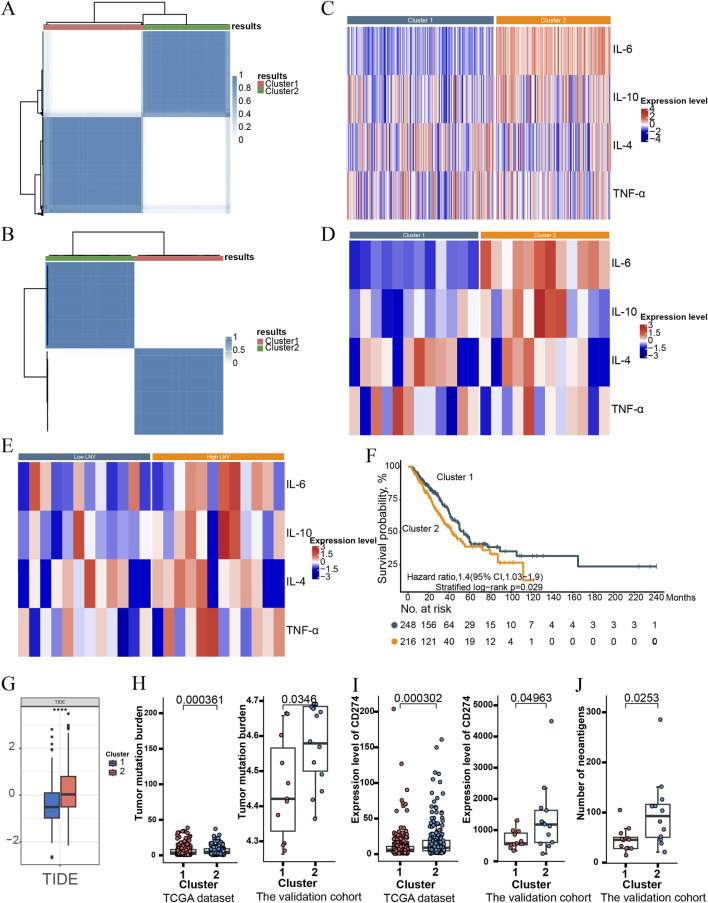
Consensus clustering of TCGA database and our validation cohort based on significantly changed immune-related molecules detected in the peripheral blood samples **(A,B)**. The heatmap showed the expression levels of immune-related molecules in Cluster 1 and 2 using TCGA database and our validation dataset **(C,D)**. The heatmap displayed the expression levels of immune-related molecules stratified by LNY using our validation dataset **(E)**. The recurrence-free survival curve of Cluster 1 and 2 in TCGA dataset **(F)**. Tumor Immune Dysfunction and Exclusion (TIDE) scores of Cluster 1 and 2 in TCGA dataset **(G)**. Tumor Mutation Burden (TMB) scores grouped by Cluster 1 and 2 in TCGA dataset and our cohort **(H)**. Expression level of CD274 grouped by Cluster 1 and 2 in TCGA dataset and our cohort **(I)**. The number of neoantigens grouped by Cluster 1 and 2 in our cohort **(J)**.

Based on the four IFCs (IL-6, IL-10, IL-4 and TNF-α), the consensus clustering algorithm identified two clusters that best represented the data pattern of LUAD populations within both TCGA dataset and our validation cohort (Figures 2A,B). Expression levels of these four IFCs stratified by the two clusters and LNY were visualized in the heatmap of Figures 2C–E. Kaplan–Meier survival plots suggested that Cluster 2 was associated with significantly poorer survival outcomes (Figure 2F). Furthermore, a significantly higher TIDE score was observed in Cluster 2 population (*p* < 0.05) (Figure 2G). As revealed in Figures 2H,I, Cluster 2 population also demonstrated increased TMB scores (*p* < 0.05) and increased expression level of CD274, similar results were corroborated in our validation cohort (Figures 2H,I). It is noteworthy that IL-6 exhibited significantly positive correlations with both TMB and TIDE (Supplementary Figure S2D). Additionally, Cluster 2 were present with higher number of neoantigens (*p* < 0.05) (Figure 2J).

**FIGURE 3:**
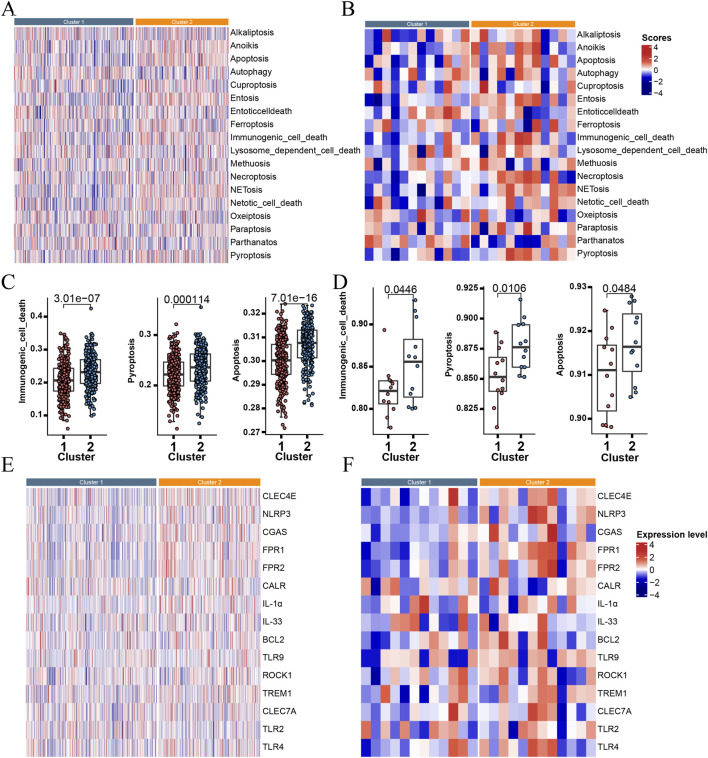
The heatmap showed cell death patterns grouped by Cluster 1 and 2 using TCGA database **(A)** and our validation dataset **(B)**. Enrichment scores of cell death profiles, including pyroptosis, immunogenic cell death and apoptosis, using TCGA database **(C)** and our validation dataset **(D)**. The heatmap showed significantly different levels in DAMPs between Cluster 1 and 2 using TCGA database **(E)** and our validation dataset **(F)**.

Enrichment scores for various cell death patterns stratified by the two clusters were displayed in Figures 3A,B, using both TCGA and our validation dataset. Among these, pyroptosis, immunogenic cell death and apoptosis were significantly enriched in Cluster 2 (Figures 3C,D) across both datasets. Finally, we further identified that Cluster 2 displayed significantly higher expression levels of DAMPs-related genes compared with Cluster 1 (Figures 3E,F) in both cohorts.

### 3.5 Bayesian meta-analysis of LNY on survival in stage I LUAD receiving SR

This Bayesian meta-analysis with the preferred reporting items for systematic reviews and meta-analyses (PRISMA) statement (Moher et al., 2009) pooled the evidence available to determine the impact of LNY on overall survival (OS) and RFS in patients with stage I LUAD underwent SR. The literature search process was shown in Supplementary Figure S3. A total of 2075 patients were included in our analysis with a median number of 196 cases. The baseline information and main characteristics are listed in Supplementary Table S3. Notably, the median cutoff value of LNY was 1 in the eligible studies. The follow-up duration was reported in five of the included studies, among which the median was 36 months. Six studies (Huang and Petersen, 2024; Wolf et al., 2011; Wang et al., 2017; Stiles et al., 2017; Cox et al., 2017; Altorki et al., 2025) involving 1770 individuals were included for the analysis of LNY and OS in resected LUAD. As shown in Supplementary Figure S4A, pooled results indicated that a greater number of LNs examined was associated with better OS (n = 6, HR = 0.74; 95%CI: 0.55–0.97) without significant heterogeneity (τ^2^ = 0.07; 95%CI: 0.00–0.49). Meanwhile, the relationships between LNY and RFS were investigated in five studies (Wolf et al., 2011; Stiles et al., 2017; Altorki et al., 2025; Chen et al., 2019; Moon et al., 2020) comprising 655 patients (Supplementary Table S3). The meta-analysis indicated that a higher LNY was not significantly associated with more improved RFS (n = 5, HR = 0.78; 95%CI: 0.56–1.05) with moderate heterogeneity observed (τ^2^ = 0.09; 95%CI: 0.00–0.62) (Supplementary Figure S4B). Subgroup analyses were performed to assess the influence of different study characteristics on these associations (Supplementary Tables S4–S5). Among patients with clinical stage I disease, a higher LNY during SR achieved better OS (HR = 0.74; 95%CI: 0.55–0.97, τ^2^ = 0.07; 95%CI: 0.00–0.68). However, there was no advantage in OS (HR = 0.76; 95%CI: 0.38–1.35, τ^2^ = 0.29; 95%CI: 0.00–2.13) in patients with p-stage I LUAD receiving a higher LNY during SR. Further subgroup analysis by different study characteristics indicated a higher LNY conferred no additional RFS benefits regardless of clinical or pathological staging in LUAD patients (HR = 0.78; 95%CI: 0.56–1.05, τ^2^ = 0.09; 95%CI: 0.00–0.62). As shown in Supplementary Figure S5A-C, there was no remarkable publication bias regarding the HRs of OS and RFS. Moreover, posterior distributions of the pooled HR were presented in Supplementary Figure S5B-D, reflecting Bayesian point and interval estimates.

## 4 Discussion

Current NCCN guidelines recommended appropriate LNs removed during SR, a minimum of 3 N2 stations sampled or a systematic LN dissection for adequate staging (Riely et al., 2024). Although the prognostic value of a higher LNY is well established in facilitating more accurate pathological staging, the therapeutic benefit of removing more lymph nodes remains debated, with conflicting evidence regarding its impact on survival. David et al. suggested harvesting 4 to 9 LNs for patients with lesions ≤ 1 cm and 10 to 16 LNs for those with lesions >1–2 cm (David et al., 2017). However, a previous analysis of SEER populations indicated that LN dissection counts ≥4 did not improve survival for subcentimeter lung cancer undergoing SR (Zheng et al., 2020). Moreover, the survival benefit of extensive LN dissection became less apparent with further increases in dissection extent, suggesting a potential double-edged sword effect of extensive LN dissection (Deng et al., 2024). On one hand, extensive LN dissection may help identify occult node metastasis, guide appropriate adjuvant treatment, and potentially improve long-term survival. On the other hand, these benefits may be counterbalanced by concerns regarding immune impairment (Lao et al., 2024). Our results suggested that fewer LNY was associated with superior RFS (HR = 0.41; 95%CI: 0.17–0.94, *p* = 0.03) in p-stage I LUAD patients received adjuvant PD-1 inhibitors after SR, but not in those undergoing SR alone (HR = 1.15; 95%CI: 0.76–1.74, *p* = 0.50). Similarly, a pooled analysis showed that a higher LNY was not associated with superior RFS (HR = 0.78; 95%CI: 0.56–1.05, τ2 = 0.09; 95%CI: 0.00–0.62). Nonetheless, subgroup analysis demonstrated that c-stage I NSCLCs with a higher LNY during SR achieved better OS (HR = 0.74; 95%CI: 0.55–0.97), as shown in Supplementary Table S4. To the contrary, more extensive LN dissection was not associated with improved OS (HR = 0.76; 95%CI: 0.38–1.35) and RFS (HR = 0.98; 95%CI: 0.20–2.94) in p-stage I LUAD.

These findings underscore the need for a nuanced approach to lymph node dissection in stage I lung cancer and highlight the importance of personalized surgical management. In particular, for pure ground-glass nodules or p-stage I NSCLC measuring ≤ 1 cm, omitted or limited LN dissection during SR might be acceptable (Lin et al., 2020). Moreover, TDLNs served as critical sites for initial tumor antigen exposure, regulating and cross-priming antitumor immune response, and were essential for T cell invigoration during checkpoint blockade therapy (Fransen et al., 2018). Accumulating evidence suggested that extensive dissection of TdLNs might lead to immune impairment, whereas TdLNs preservation could enhance immunotherapy efficacy by maintaining antigen-specific immune activation (Fransen et al., 2018; Munn and Mellor, 2006). By evaluating IFC dynamics and integrating data from our institutional cohort with meta-analytic findings, this study sheds light on the potential immune mechanisms underlying these associations. When comparing IFCs in the higher LNY group with those in the lower one before and after surgery, we observed more pronounced increases in IL-6, IL-10, IL-4, and TNF-α in the higher LNY group, suggesting SR with a lower LNY might induce a mild inflammatory response and cause less non-specific immune damage compared to more extensive LN removal. Supporting this, another study assessing immune response of their peripheral blood showed that selective LN dissection resulted in less impairment of cellular immune function than the systematic LN dissection in early-stage NSCLCs (Zhao et al., 2021). Recent advances have demonstrated IL-6 as a pivotal regulator of the tumor microenvironment and therapy response (Ingram et al., 2022; He et al., 2025). Elevated postoperative IL-6 levels might reprogram the immune landscape by driving tumor-associated macrophage polarization towards a pro-tumorigenic M2 phenotype, potentially contributing to resistance against subsequent immunotherapy (He et al., 2025). Thus, although extensive LN dissection could improve staging accuracy, excessive removal of TdLNs might have long-lasting adverse effects on systemic anti-tumor immunity, which might contribute to tumor resistance mechanisms with inadequate T cell priming (Shen et al., 2026), particularly in patients receiving adjuvant immunotherapy.

Employing unsupervised clustering methods on LN dissection-related IFCs (Song et al., 2024), we further identified a subpopulation of LUAD patients who might benefit from adjuvant PD-1 blockade. The two clusters represented clinically distinct subgroups in immune and cell death profiles with regard to RFS. The Cluster 2 exhibited higher TIDE and TMB scores, along with elevated CD274 expression, suggesting that these patients might experience greater benefits from ICIs. These findings were corroborated in an independent validation cohort of 24 patients, in which Cluster 2 subgroup similarly demonstrated higher TMB, increased CD274 expression, distinct cell death profiles, and further showed a higher number of neoantigens, indicating that these individuals represent potential candidates for adjuvant PD-1/PD-L1 inhibitors. However, the impact of these cytokine responses on the long-term efficacy of subsequent immunotherapy remains to be further elucidated.

Several limitations of this study should be acknowledged. First, the relatively small size of the validation cohort may have limited the robustness of the consensus clustering findings. Second, the follow-up duration for patients undergoing SR in our cohort was insufficient to assess long-term outcomes. While the current findings primarily reflect early-to-mid-term risk in recurrence, extended follow-up is warranted to determine the long-term overall survival in this patient population. Third, although a blinded pathological review was conducted, the assessment of the number of lymph nodes remained subject to inter-observer variability. Differences in histological interpretation of nodal tissue might affect the accuracy of nodal counts and staging. Fourth, the cohort included patients with varying PD-L1 expression levels (negative, weakly, and strongly). Since patients might exhibit differential responses to ICIs, this heterogeneity probably influenced the observed treatment outcomes. Finally, inherent selection bias was inevitable due to the retrospective nature of the data analysis.

## 5 Conclusion

In conclusion, available evidence indicates that increased LNY during SR might not confer significant improvements in RFS among patients with p-stage I lung cancer. Excessive removal of LNs might adversely affect physiological responsiveness to immunochemotherapy. Personalized lymph node management should be considered for appropriately selected patients with node-negative disease.

## Data Availability

The original contributions presented in the study are publicly available. The next-generation sequencing (NGS) and whole exome sequencing (WES) data can be accessed through the Genome Sequence Archive (GSA-Human) at the National Genomics Data Center (https://ngdc.cncb.ac.cn/gsa-human). under accession numbers HRA017281 and HRA017282.
