# Supplementary material for: Prognostic impact of the lymph node yield on survival in patients with stage I lung adenocarcinoma receiving sublobar resection
Source: Front Mol Biosci. 2026 Mar 24;13:1727569. doi: 10.3389/fmolb.2026.1727569 (PMC13053242; doi:10.3389/fmolb.2026.1727569)
Supplement: Supplementary file 1 [file Supplementaryfile1.docx]

| Supplementary Table 1. The clinicopathological characteristics of LUAD tissues in TCGA (n = 464). | | |
| --- | --- | --- |
| Variable | Cluster1 | Cluster2 |
| **Number** | 248 | 216 |
| **Gender** |  |  |
| Male | 110 | 103 |
| Female | 138 | 113 |
| **Age( years)** |  |  |
| ≥65 | 133 | 121 |
| ＜65 | 110 | 93 |
| NA | 5 | 2 |
| **Smoking history** |  |  |
| ≥30 | 51 | 40 |
| ＜30 | 45 | 43 |
| NA | 152 | 133 |
| **Operation** |  |  |
| Lobectomy | 245 | 212 |
| Other | 3 | 4 |
| **Pathological stage** |  |  |
| I | 144 | 109 |
| II | 53 | 56 |
| III | 37 | 41 |
| IV | 14 | 10 |
| **T stage** |  |  |
| T1 | 91 | 60 |
| T2 | 124 | 130 |
| T3 | 22 | 17 |
| T4 | 11 | 9 |
| **N stage** |  |  |
| N0 | 167 | 134 |
| N1 | 42 | 46 |
| N2 | 30 | 32 |
| N3 | 1 | 1 |
| NA | 8 | 3 |
| **M stage** |  |  |
| M0 | 159 | 152 |
| M1 | 13 | 10 |
| NA | 76 | 54 |
| Abbreviations: LUAD, lung adenocarcinoma; NR, not reported. | | |

| Supplementary Table 2. The clinicopathological characteristics of LUAD tissues of our dataset (n = 24). | | |
| --- | --- | --- |
| Variable | Cluster1 | Cluster2 |
| **Number** | 12 | 12 |
| **Gender** |  |  |
| Male | 6 | 7 |
| Female | 6 | 5 |
| **Age( years)** |  |  |
| ≥65 | 5 | 6 |
| ＜65 | 7 | 6 |
| **Smoking history** |  |  |
| Yes | 3 | 4 |
| No | 9 | 8 |
| **Operation** |  |  |
| Lobectomy | 7 | 6 |
| Sublobar Resection | 5 | 6 |
| **LNY** |  |  |
| High (≥4) | 6 | 6 |
| Low (＜4) | 6 | 6 |
| **Pathological stage** |  |  |
| I | 12 | 12 |
| Abbreviations: LUAD, lung adenocarcinoma; LNY, lymph node yield. | | |

| Supplementary Table 3. Baseline characteristics of included studies investigating the association between LNY during sublobar resection for stage I LUADs (n = 8) | | | | | | | | | | | | | | | | | |
| --- | --- | --- | --- | --- | --- | --- | --- | --- | --- | --- | --- | --- | --- | --- | --- | --- | --- |
| No. | Authors | Country | Inclusion period | Study design | Number of cases (F/M) | Age (years) (range) | Clinical stage | Pathological stage | Surgical procedure: (wedge resection/segmentectomy) | Histology | Number of examined LN | LN cutoff value | Number of patients stratified by LN cutoff value (high/low) | Follow-up period (months) | Weight distribution (%) | Analysis of hazard ratio | Quality score |
| 1 | Wang Y et al (2017) [30] | China | 2010-2015 | Retrospective study | 746 (456/290) | NR | IA | IA | Wedge: 746 | LUAD | NR | 1 | 285/461 | 18.96 (0.07-71.7) | OS: 15.69 | Univariate | 7 |
| 2 | Stiles BM et al (2015) [31] | USA | 2000-2014 | Retrospective study | 196 (119/77) | 73 (67-69) | IA | IA-IIIB | Wedge: 196 | LUAD/Other | 4 | 1 | 138/58 | 35 (24-78) | OS: 7.57, RFS: 13.58 | Multivariate | 8 |
| 3 | Wolf AS et al (2011) [29] | USA | 2000-2005 | Retrospective study | 154 (92/62) | 71 (41-91) | IA | IA-IIB | Wedge/Seg: 130/24 | LUAD/Other | NR | 1 | 45/109 | NR | OS:24.08, RFS: 25.20 | Univariate | 7 |
| 4 | Chen C et al (2019) [34] | China | 2010-2014 | Retrospective study | 112 (53/59) | 77 (75-85) | IA | IA-B | Wedge/Seg: NR | LUAD | NR | 5 | 11/101 | 60.2 (1.3-99.8) | RFS: 16.14 | Univariate | 7 |
| 5 | Moon Y et al (2020) [35] | Korea | 2008-2017 | Retrospective study | 193 (117/76) | 63.9 | IA-B | IA-B | Wedge/seg:101/92 | LUAD | 5.5 (±6.9) | NR | NR | 36.0 (4.9-108.6) | RFS: 29.86 | Univariate | 7 |
| 6 | Cox ML et al (2017) [32] | USA | 2003-2006 | Retrospective study | 447 (308/139) | 70 (62-77) | I | IA-IIIB | Wedge/seg:NR | LUAD | NR | 1 | 199/248 | NR | OS: 27.53 | Univariate | 7 |
| 7 | Huang L et al (2024) [27] | Denmark | 2008-2023 | Retrospective study | 227 (153/74) | 71 (64-76) | IA-B | IA-IIIB | Seg:227 | LUAD/ Other | 9 (6–12) | 9 | 96/131 | 38.4 | OS: 11.42 | Univariate | 8 |
| 8 | Altorki N et al (2025) [33] | Canada. | 2007-2017 | Randomized study | 336 | NR | IA | IA | Wedge/seg: 147/131 | LUAD/ Other | NR | NR | 86/250 | 84 | OS:13.71, RFS: 15.22 | Univariate | 8 |
| Abbreviations: NR, not reported; Seg, segmentectomy; LUAD, lung adenocarcinoma; LN, lymph node; LNY, lymph node yield; OS, overall survival; RFS, recurrence-free survival. | | | | | | | | | | | | | | | | | |

| Supplementary Table 4. Subgroup analyses of the associations between LNY and OS (n = 6) | | | | | | |  |
| --- | --- | --- | --- | --- | --- | --- | --- |
| Variables | Number of studies | Test of association | |  | Test of heterogeneity | | |
|  |  | HR | 95% CI |  | τ^2^ | 95% CI | |
| Total | 6 | 0.74 | 0.55-0.97 |  | 0.07 | 0.00-0.49 | |
| **Publication year** |  |  |  |  |  |  | |
| ≥2017 | 4 | 0.82 | 0.51-1.35 |  | 0.19 | 0.00-1.65 | |
| <2017 | 2 | 0.76 | 0.12-2.37 |  | 0.92 | 0.00-3.66 | |
| **Initial inclusion period** |  |  |  |  |  |  | |
| ≥2005 | 3 | 0.95 | 0.40-2.01 |  | 0.40 | 0.00-2.95 | |
| <2005 | 3 | 0.69 | 0.26-1.41 |  | 0.41 | 0.00-2.88 | |
| **Research region** |  |  |  |  |  |  | |
| China | 1 | 0.68 | 0.43-1.08 |  | -- | -- | |
| USA | 3 | 0.69 | 0.28-1.32 |  | 0.39 | 0.00-2.79 | |
| Denmark | 1 | 0.92 | 0.52-1.61 |  | -- | -- | |
| Canada | 1 | 1.06 | 0.64-1.75 |  | -- | -- | |
| **Number of cases** |  |  |  |  |  |  | |
| ≥200 | 4 | 0.81 | 0.49-1.34 |  | 0.20 | 0.00-1.56 | |
| <200 | 2 | 0.81 | 0.13-2.85 |  | 0.94 | 0.00-3.62 | |
| **Median age (years)** |  |  |  |  |  |  | |
| ≥71 | 3 | 0.74 | 0.26-1.66 |  | 0.51 | 0.00-3.05 | |
| <70 | 1 | 0.68 | 0.51-0.92 |  | -- | -- | |
| NR | 2 | 1.08 | 0.20-3.54 |  | 0.84 | 0.00-3.59 | |
| **Study design** |  |  |  |  |  |  | |
| Retrospective | 5 | 0.71 | 0.49-0.96 |  | 0.09 | 0.00-0.68 | |
| Randomized | 1 | 1.06 | 0.64-1.75 |  | -- | -- | |
| **Tumor stage** |  |  |  |  |  |  | |
| Clinical stage I | 6 | 0.74 | 0.55-0.97 |  | 0.07 | 0.00-0.49 | |
| Pathological stage I | 4 | 0.76 | 0.38-1.35 |  | 0.29 | 0.00-2.13 | |
| **Histology** |  |  |  |  |  |  | |
| LUAD | 2 | 0.87 | 0.18-2.48 |  | 0.67 | 0.00-3.45 | |
| LUAD/Other | 4 | 0.81 | 0.41-1.42 |  | 0.32 | 0.00-2.25 | |
| **Surgical procedure** |  |  |  |  |  |  | |
| Segmentectomy/wedge resection | 3 | 0.86 | 0.41-1.69 |  | 0.34 | 0.00-2.70 | |
| Wedge resection | 2 | 0.74 | 0.11-2.33 |  | 0.87 | 0.00-3.66 | |
| Segmentectomy | 1 | 0.92 | 0.52-1.61 |  | -- | -- | |
| **LN cutoff value** |  |  |  |  |  |  | |
| >1 | 1 | 0.92 | 0.52-1.61 |  | -- | -- | |
| 1 | 4 | 0.68 | 0.45-0.96 |  | 0.16 | 0.00-1.18 | |
| NR | 1 | 1.06 | 0.64-1.75 |  | -- | -- | |
| **Follow-up period (months)** |  |  |  |  |  |  | |
| ≥30 | 3 | 0.89 | 0.27-2.24 |  | 0.67 | 0.00-3.18 | |
| <30 | 1 | 0.68 | 0.43-1.08 |  | -- | -- | |
| NR | 2 | 0.86 | 0.18-2.63 |  | 0.62 | 0.00-3.46 | |
| **Populations** |  |  |  |  |  |  | |
| Eastern | 1 | 0.68 | 0.43-1.08 |  | -- | -- | |
| Western | 5 | 0.76 | 0.51-1.08 |  | 0.13 | 0.00-1.01 | |
| **Quality score** |  |  |  |  |  |  | |
| 7 | 3 | 0.73 | 0.39-1.25 |  | 0.21 | 0.00-1.89 | |
| 8 | 3 | 0.92 | 0.26-2.50 |  | 0.67 | 0.00-3.18 | |
| **Analysis of hazard ratio** |  |  |  |  |  |  | |
| Multivariate | 1 | 0.44 | 0.21-0.89 |  | -- | -- | |
| Univariate | 5 | 0.79 | 0.59-1.06 |  | 0.07 | 0.00-0.48 | |
| Abbreviations: NR, not reported; LUAD, lung adenocarcinoma; LN, lymph node; LNY, lymph node yield; OS, overall survival; HR, hazard ratio; CI, confidence interval. | | | | | | | |

| Supplementary Table 5. Subgroup analyses of the associations between LNY and RFS (n=5) | | | | | |  |
| --- | --- | --- | --- | --- | --- | --- |
| Variables | Number of studies | Test of association | |  | Test of heterogeneity | |
|  |  | HR | 95% CI |  | τ^2^ | 95% CI |
| Total | 5 | 0.78 | 0.56-1.05 |  | 0.09 | 0.00-0.62 |
| **Publication year** |  |  |  |  |  |  |
| ≥2017 | 3 | 0.90 | 0.49-1.62 |  | 0.23 | 0.00-2.07 |
| <2017 | 2 | 0.80 | 0.14-2.46 |  | 0.84 | 0.00-3.55 |
| **Initial inclusion period** |  |  |  |  |  |  |
| ≥2005 | 3 | 0.90 | 0.49-1.62 |  | 0.23 | 0.00-2.07 |
| <2005 | 2 | 0.80 | 0.14-2.46 |  | 0.84 | 0.00-3.55 |
| **Research region** |  |  |  |  |  |  |
| China | 1 | 0.82 | 0.52-1.30 |  | -- | -- |
| USA | 2 | 0.83 | 0.14-2.59 |  | 0.86 | 0.00-3.60 |
| Korea | 1 | 0.82 | 0.62-1.08 |  | -- | -- |
| Canada | 1 | 0.92 | 0.57-1.48 |  | -- | -- |
| **Number of cases** |  |  |  |  |  |  |
| ≥160 | 3 | 0.81 | 0.31-1.79 |  | 0.49 | 0.00-3.02 |
| <160 | 2 | 0.93 | 0.22-2.73 |  | 0.64 | 0.00-3.39 |
| **Median age (years)** |  |  |  |  |  |  |
| ≥71 | 2 | 0.82 | 0.16-2.84 |  | 0.88 | 0.00-3.51 |
| <71 | 2 | 0.98 | 0.23-2.91 |  | 0.61 | 0.00-3.28 |
| NR | 1 | 0.92 | 0.57-1.48 |  | -- | -- |
| **Study design** |  |  |  |  |  |  |
| Retrospective | 4 | 0.76 | 0.45-1.12 |  | 0.18 | 0.00-1.60 |
| Randomized | 1 | 0.92 | 0.57-1.48 |  | -- | -- |
| **Tumor stage** |  |  |  |  |  |  |
| Clinical stage I | 5 | 0.78 | 0.56-1.05 |  | 0.09 | 0.00-0.62 |
| Pathological stage I | 3 | 0.98 | 0.20-2.94 |  | 0.66 | 0.00-3.48 |
| **Histology** |  |  |  |  |  |  |
| LUAD | 2 | 1.00 | 0.21-2.89 |  | 0.63 | 0.00-3.35 |
| LUAD/Other | 3 | 0.81 | 0.29-1.97 |  | 0.49 | 0.00-3.07 |
| **Surgical procedure** |  |  |  |  |  |  |
| Segmentectomy/wedge resection | 4 | 0.83 | 0.60-1.12 |  | 0.08 | 0.00-0.68 |
| Wedge resection | 1 | 0.50 | 0.30-0.84 |  | -- | -- |
| **LN cutoff value** |  |  |  |  |  |  |
| >1 | 1 | 0.82 | 0.52-1.30 |  | -- | -- |
| 1 | 2 | 0.81 | 0.14-2.80 |  | 0.82 | 0.00-3.52 |
| NR | 2 | 0.97 | 0.25-2.87 |  | 0.58 | 0.00-3.32 |
| **Follow-up period (months)** |  |  |  |  |  |  |
| ≥50 | 2 | 1.03 | 0.24-2.97 |  | 0.69 | 0.00-3.42 |
| <50 | 2 | 0.84 | 0.14-2.81 |  | 0.92 | 0.00-3.62 |
| NR | 1 | 0.77 | 0.56-1.07 |  | -- | -- |
| **Populations** |  |  |  |  |  |  |
| Eastern | 2 | 1.04 | 0.23-3.21 |  | 0.64 | 0.00-3.45 |
| Western | 3 | 0.78 | 0.29-1.59 |  | 0.49 | 0.00-3.00 |
| **Quality score** |  |  |  |  |  |  |
| 7 | 3 | 0.84 | 0.43-1.49 |  | 0.21 | 0.00-1.95 |
| 8 | 2 | 0.91 | 0.13-3.10 |  | 0.96 | 0.00-3.57 |
| **Analysis of hazard ratio** |  |  |  |  |  |  |
| Multivariate | 1 | 0.50 | 0.30-0.84 |  | -- | -- |
| Univariate | 4 | 0.78 | 0.45-1.22 |  | 0.18 | 0.00-1.51 |
| Abbreviations: NR, not reported; LUAD, lung adenocarcinoma; LN, lymph node; LNY, lymph node yield; RFS, recurrence-free survival; HR, hazard ratio; CI, confidence interval. | | | | | | |
|  | | | | | | |
|  | | | | | | |
