# Supplementary figures and images for: Prognostic impact of the lymph node yield on survival in patients with stage I lung adenocarcinoma receiving sublobar resection
Source: Front Mol Biosci. 2026 Mar 24;13:1727569. doi: 10.3389/fmolb.2026.1727569 (PMC13053242; doi:10.3389/fmolb.2026.1727569)

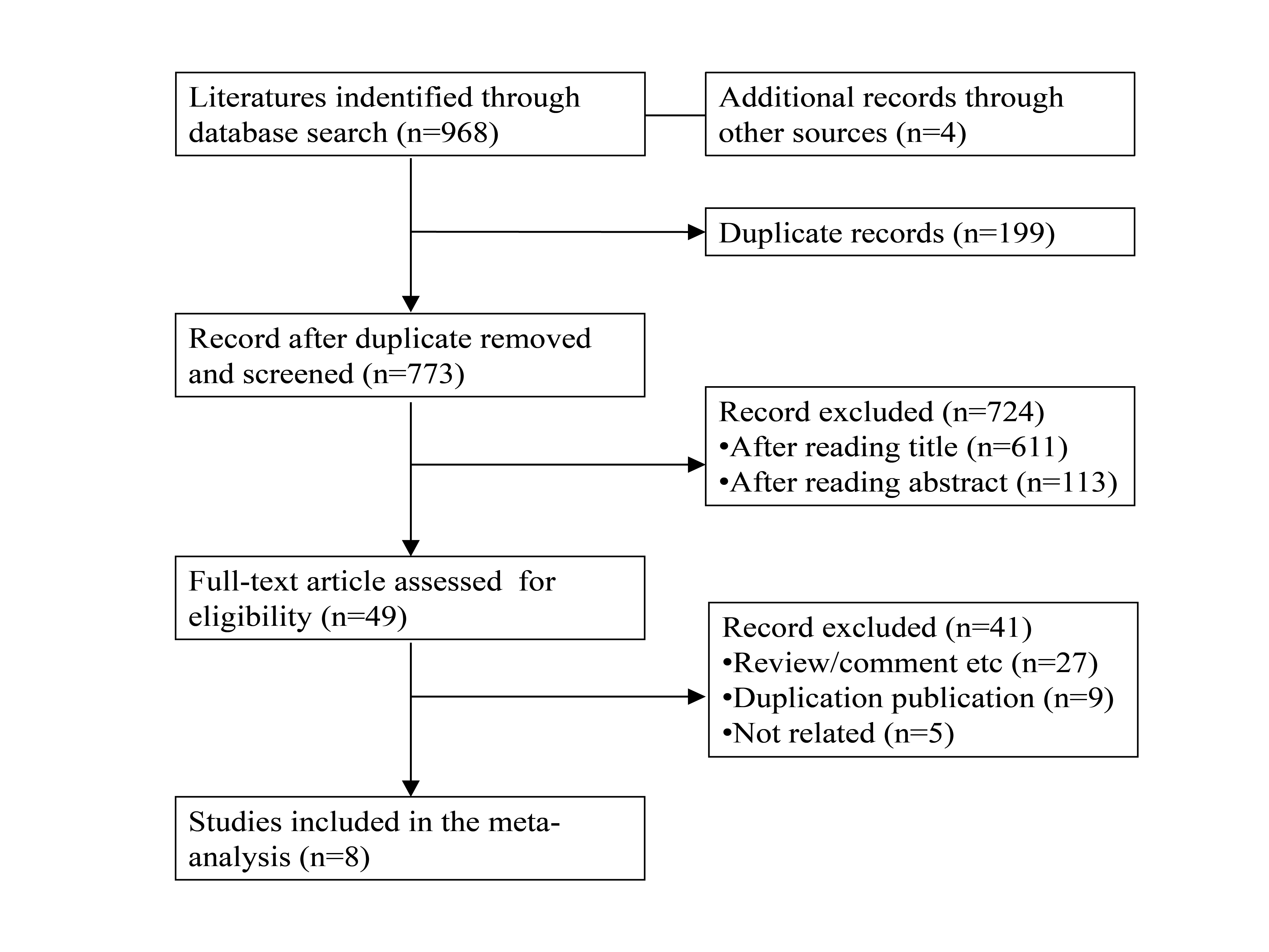

Supplement: Supplementary file 2 [file Image3.tif]

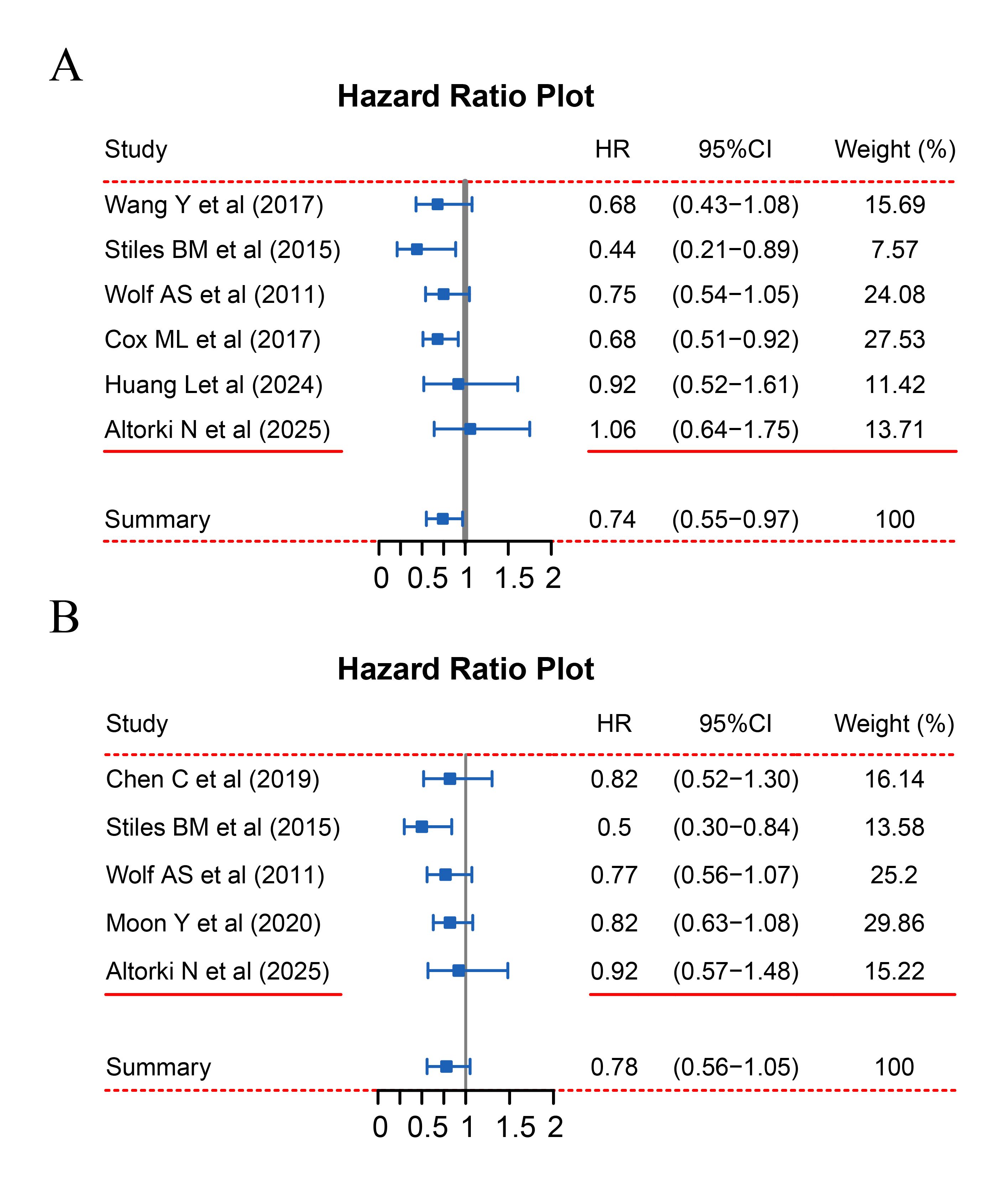

Supplement: Supplementary file 3 [file Image4.tif]

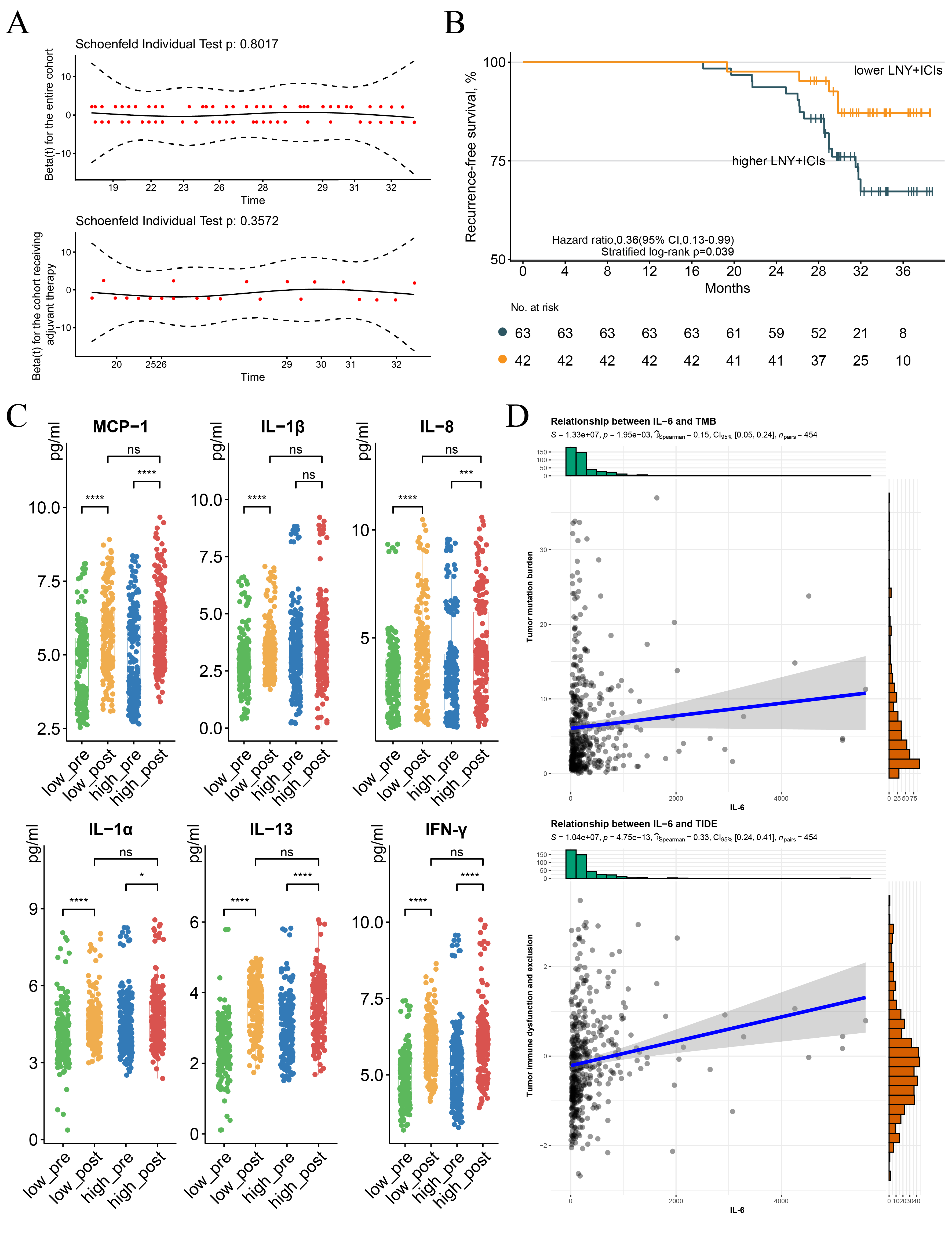

Supplement: Supplementary file 5 [file Image2.tif]

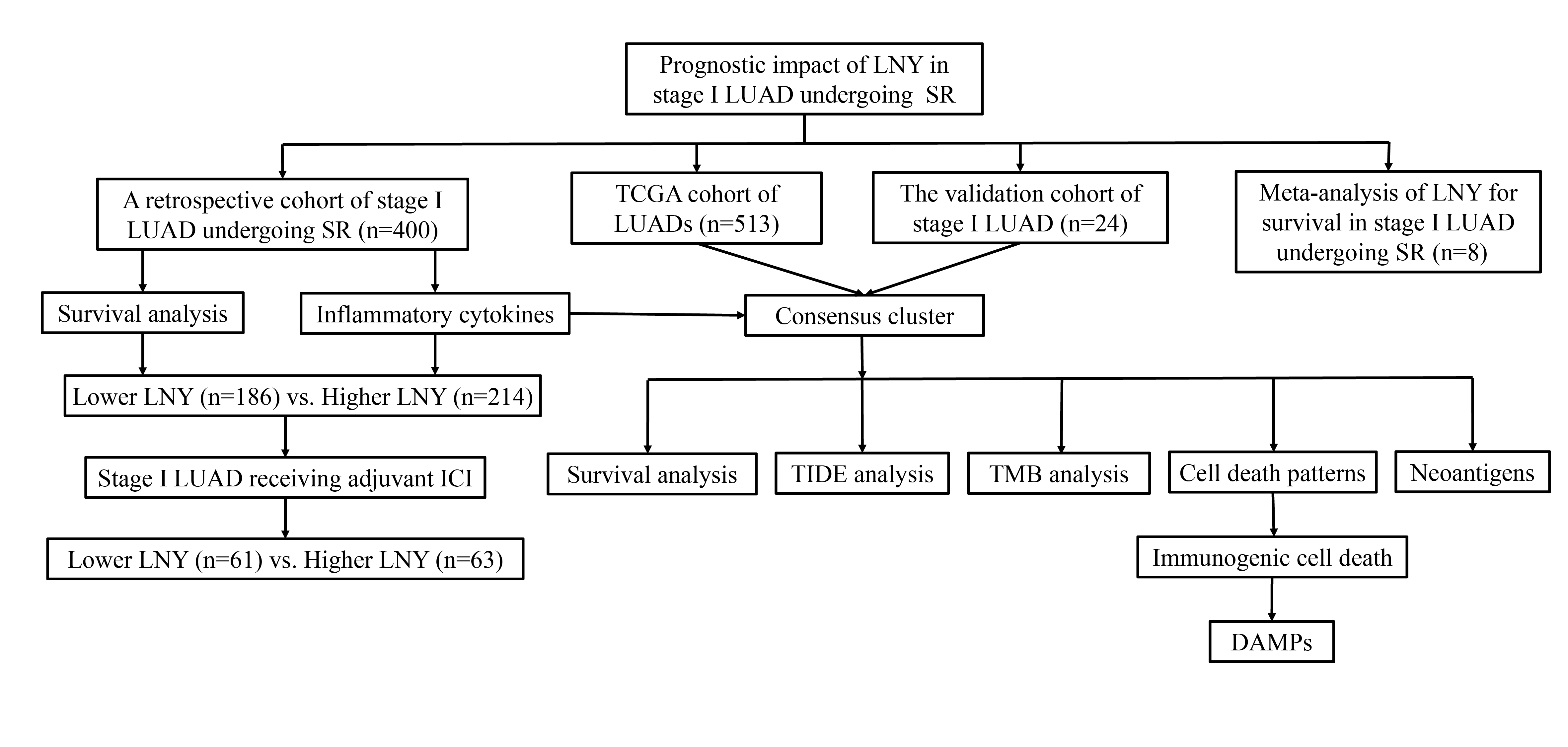

Supplement: Supplementary file 6 [file Image1.tif]

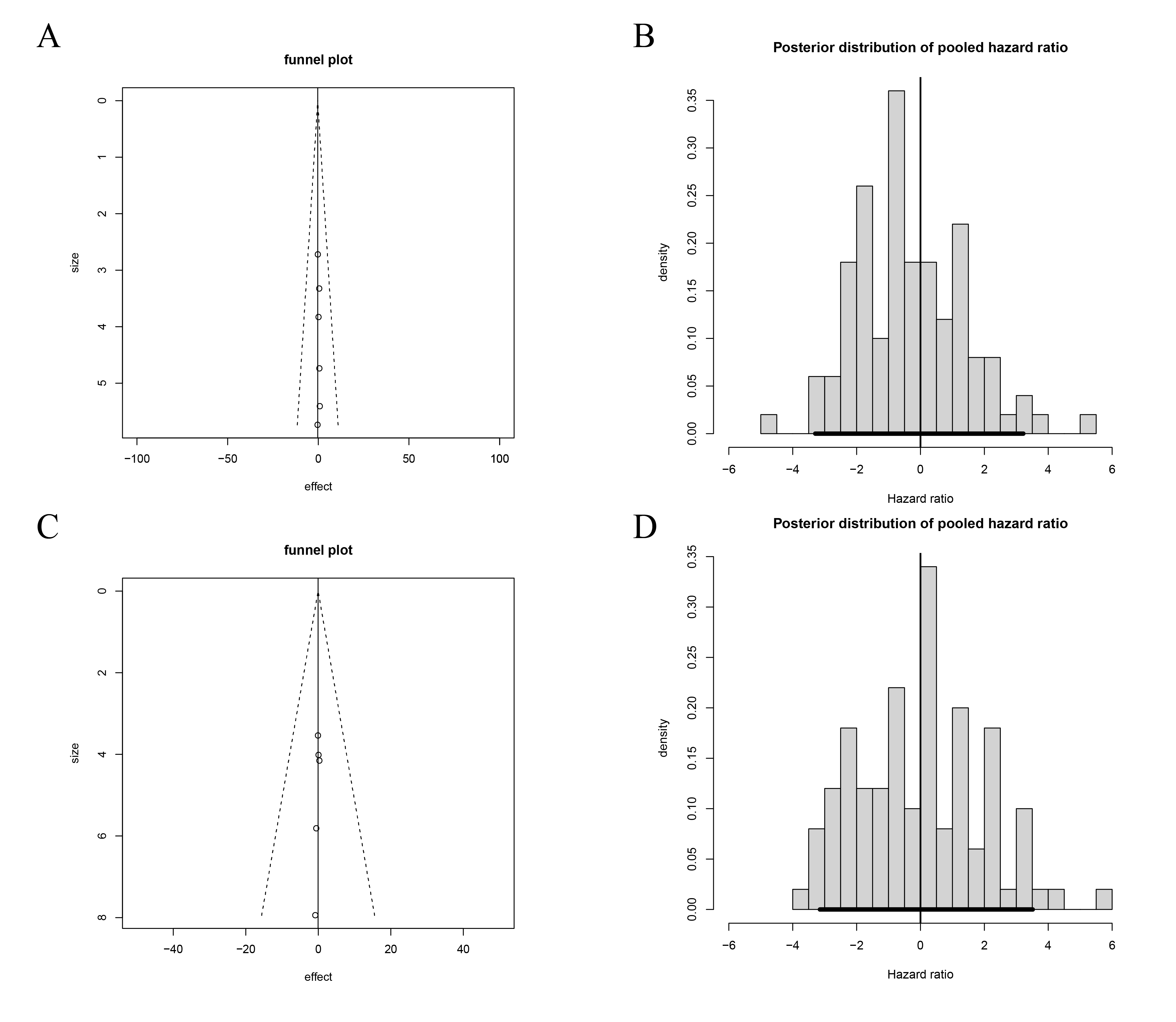

Supplement: Supplementary file 7 [file Image5.tif]
